# Knowledge, attitude, and perception regarding the respiratory syncytial virus vaccine among healthcare professionals

**DOI:** 10.1080/20523211.2025.2482669

**Published:** 2025-03-28

**Authors:** Khawla Abu Hammour, Qusai Al Manaseer, Mariam Abdel-Jalil, Faris El-dahiyat, Walid Abu Hammour, Adnan M. Abu Hammour, Fahmi Y. Al-Ashwal, Rana Abu-Farha

**Affiliations:** a Department of Clinical Pharmacy and Biopharmaceutics, Faculty of Pharmacy, University of Jordan, Amman, Jordan; b Department of Orthopedic, Faculty of Medicine, University of Jordan, Amman, Jordan; c College of Pharmacy, Al Ain University, Abu Dhabi, UAE; d School of Medicine, University of Jordan, Amman, Jordan; e College of Medicine, Mohammed Bin Rashid University of Medicine and Health Science, Dubai, UAE; f Department of Clinical Pharmacy and Pharmacy Practice, Faculty of Pharmacy, University of Science and Technology, Sana'a, Yemen; g Department of Clinical Pharmacy, College of Pharmacy, Al-Ayen Iraqi University, Thi-Qar, Iraq; h Department of Clinical Pharmacy and Therapeutics, Faculty of Pharmacy, Applied Science Private University, Amman, Jordan

**Keywords:** Respiratory syncytial virus, vaccine, knowledge, perception, healthcare providers

## Abstract

**Background:**

Respiratory syncytial virus (RSV) is a significant respiratory pathogen. Despite vaccine availability, uptake remains low, and healthcare professionals play a key role in promoting immunisation. This study aims to evaluate healthcare providers' knowledge, perceptions and practices regarding the RSV vaccine.

**Methods:**

A validated survey was distributed to healthcare professionals. The study questionnaire contained sections to assess sociodemographic characteristics, knowledge of RSV and its vaccines, healthcare professionals' perception towards RSV and its vaccines, and their perception towards the potential barriers against RSV vaccination. The last section assesses physicians' previous practice in dealing with RSV infection.

**Results:**

Over half of the participants (56.6%) had no prior awareness of RSV, though many recognised its potential severity, particularly in vulnerable populations like children and older adults (52.6%). Awareness of FDA-approved RSV vaccines was limited, with only 28.1% of respondents familiar with vaccines intended for older adults. Views on vaccination recommendations for older age groups were divided; 23.7% supported vaccination for those 75 and older, while 31.1% advocated for vaccination in those aged 60-74 at higher risk. Perceived barriers to RSV vaccination were prominent. Most respondents (85.1%) cited concerns about vaccine safety as a key obstacle, and 81.1% identified out-of-pocket costs as a significant barrier. Testing for RSV was infrequent (24.6%), mainly due to a lack of effective treatment. Most participants (96.9%) called for greater awareness and education about RSV vaccines, and 91.2% supported recommending the vaccine if it was available and free.

**Conclusion:**

This study reveals significant gaps in healthcare professionals' knowledge and practices regarding RSV and its vaccines, with substantial barriers to vaccine adoption. Targeted education, improved diagnostics, and addressing vaccine barriers are essential strategies for improving the healthcare response to RSV.

## 1. Background

Respiratory Syncytial Virus (RSV) is a major global respiratory pathogen that affects individuals across all age groups, with particularly severe consequences for infants, young children, and older adults. It is a leading cause of respiratory illness worldwide, contributing significantly to the burden of hospitalisations and morbidity, especially during seasonal outbreaks. RSV's life cycle depends on its surface proteins, G and F, which enable it to adhere to and fuse with host cells. The virus is mostly spread by respiratory droplets and contaminated surfaces (CDC, 2024).

Since RSV infections frequently have nonspecific signs and symptoms, it can be difficult to diagnose them early, which can cause delays in treatment and raise the risk of serious consequences such pneumonia, cute respiratory distress, and respiratory failure (Hall, 2001; Lee et al., 2019). Additionally, RSV can worsen comorbidities like asthma and chronic obstructive pulmonary disease (COPD) because to its regular seasonal transmission pattern, which usually peaks in the winter (CDC, 2024).

The RSV’s seasonal trends have been notably influenced by the COVID-19 pandemic, contributing to a resurgence of infections because of reduced viral exposures during lockdowns which resulted in lowering the immunity levels (Bhardwaj et al., 2024). This pattern highlights RSV as an important, although sometimes underestimated, threat of public health, particularly among the elderly.

In the United States, RSV-related hospitalisations are estimated to range between 123,000 and 193,000 annually (Havers et al., 2024). While monoclonal antibody treatments are available for high-risk children, no comparable therapies exist for older populations, underscoring the urgent need for preventive measures like vaccination, along with improved diagnostic testing and hygiene protocols.

Recently, significant progress has been made in RSV vaccine development. On 3 May 2023, the U.S. Food and Drug Administration (FDA) approved Arexvy, the first RSV vaccine for individuals aged 60 years and older, to prevent lower respiratory tract disease associated with RSV (FDA, 2023). This approval marks an important step in addressing RSV-related health risks, in particular for vulnerable populations.

Despite these advancements, there is a substantial gap in understanding the knowledge, attitudes, and perceptions of healthcare professionals (HCPs) regarding RSV vaccination, which is essential for increasing vaccine uptake across communities (Kherfan & Sallam, 2023).

Vaccination is one of the most valuable public health interventions, contributing to the reduction or eradication of several life-threatening infectious diseases globally (Kaur, 2024). The World Health Organization estimates that immunisation prevents between 3.5 and 5 million deaths annually worldwide (WHO, 2024). However, despite the proven benefits of vaccines, there remain significant barriers to vaccination, often fuelled by public perception of vaccine risks. Research has shown that trust in public health authorities plays a key role in individuals’ willingness to vaccinate (Goje & Kapoor, 2024). Furthermore, the introduction of new vaccines strengthens health systems by enhancing the knowledge and skills of healthcare professionals, who play a crucial role in educating the public about vaccine safety and efficacy (de Koning et al., 2024).

Given the critical role of healthcare professionals in both directly treating patients and disseminating information about vaccination, it is essential to assess their knowledge, attitudes, and perceptions regarding RSV immunisation. Healthcare professionals are more likely to recommend vaccines to patients and the broader community if they themselves are informed and supportive of vaccination. This, in turn, increases vaccine uptake and improves public health outcomes. For the general population, widespread adoption of RSV vaccination can significantly reduce the health burden associated with RSV infections, particularly in vulnerable groups such as infants, the elderly, and immunocompromised individuals.

While global efforts have focused on improving vaccination coverage, there is limited research on the specific knowledge and attitudes of healthcare professionals toward RSV vaccination in different regions. Most studies on this topic focus on Europe (Ponticelli et al., 2024), and few address the unique context of the Middle East. In Jordan, for example, there is a lack of studies examining healthcare professionals’ intentions to recommend RSV vaccination, despite the significant potential impact on public health in the region. This gap in the literature is particularly notable as Jordan’s National Vaccines Committee is currently considering new guidelines for RSV vaccination, making it timely to evaluate healthcare professionals’ knowledge, attitudes, and anticipated practices regarding RSV immunisation.

This study, therefore, aims to assess the knowledge, attitudes, and anticipated vaccination practices among healthcare professionals in Jordan concerning the introduction of new RSV vaccination guidelines. Understanding these factors is crucial for guiding public health policies and ensuring that healthcare workers are equipped to promote RSV vaccination effectively.

## 2. Methods

### 2.1. Study design, sample, and setting

This research is a cross-sectional study aimed to assess healthcare professionals’ knowledge, perceptions, and practices regarding RSV and its vaccines in Jordan. The study was conducted among healthcare professionals in Jordan between August and October 2024.

### 2.2. Data collection

An electronic survey was distributed to a convenience sample of healthcare professionals in Jordan via social media platforms, including Facebook and WhatsApp. Before participation, respondents received a comprehensive explanation of the study's objectives and were assured of their responses’ voluntary nature and anonymity.

### 2.3. Survey development

**Figure 1.**
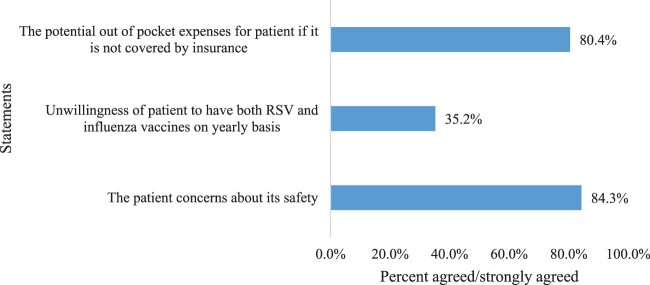
Potential barriers against RSV vaccination as perceived by the respondents (*n* = 228).

An extensive literature review informed the development of the questionnaire on RSV and its vaccines (Ackerson et al., 2019; Drysdale & Broadbent, 2023; Farquharson et al., 2024; Houle & Andrew, 2024; Ruckwardt, 2023). The questionnaire underwent content and face validity validation by two clinical pharmacy experts. It was reviewed for content validity by them. They reviewed the questionnaire and suggested minor modification to the original questionnaire. The first section covers demographic characteristics (e.g. age, gender, specialisation, and years of experience). The second section comprises closed questions assessing healthcare professionals’ awareness, perceptions, and respondents’ practice regarding RSV and RSV vaccines. In the practice section, the participants were asked, for example, if they provided care for RSV patients, checked the presence of RSV infection in patients with respiratory tract infection (Table 2). The third section evaluates healthcare professionals’ perception towards the potential barriers against RSV vaccination (Figure 1), and the fourth section assesses healthcare professionals’ knowledge of RSV and its vaccines (Table 3). A pilot study was carried out with five healthcare professionals to evaluate the questionnaire’s format, clarity, length, reliability, and general impression before it was distributed to the full sample Following their feedback, modifications were made to some of the questions. The data from this pilot study were excluded from the final analysis.

The internal consistency of the questionnaire was confirmed using Cronbach's alpha for sections two and four, showing satisfactory reliability of the items in these sections, with values of 0.722 and 0.882, respectively (Taber, 2018). Knowledge questions were scored with a value of 1 for correct answers and 0 for incorrect answers. A total score range of (0–9) for each participant.

### 2.4. Sample size determination

Adhering to Tabachnick and Fidell guidelines for sample size calculation while conducting regression analysis, 5–20 subjects are required per each predictor (Tabachnick & Fidell, 2006). Using the upper limit and since five predictors are hypothetically according to previous researches assumed to influence healthcare professionals’ responses in the current study, the minimum sample size required to ensure satisfactory statistical power was found to be equal to 100 participants. The five predictors were selected based on prior research and theoretical frameworks regarding health behaviour and vaccination decision-making in healthcare professionals (Leigh et al., 2022). These predictors have been identified in previous studies examining healthcare professionals’ attitudes toward vaccination, including factors such as knowledge, attitudes, perceptions, professional role, and experience with RSV-related clinical cases.

### 2.5. Ethical considerations

Ethical approval was obtained from the Institutional Review Board (IRB) of Jordan University Hospital (10/2024/8258), and electronic informed consent was secured from each participating healthcare professional. The study strictly adhered to the ethical standards outlined in the Declaration of Helsinki.

### 2.6. Statistical analysis

Data were analyzed using SPSS version 22.0 (IBM Corp, Armonk, NY). Categorical variables were presented as frequencies and percentages. Initial screening of independent variables affecting knowledge scores regarding RSV vaccines was performed using simple linear regression, including variables with a *p*-value of less than 0.25 in subsequent multiple linear regression analysis, ensuring independence and absence of multicollinearity. Statistical significance was set at *p*-value < 0.05.

## 3. Results

The study comprised 228 healthcare professionals, with 16.7% of participants aged more than 36 years old (*n* = 38). Of the respondents, 74.1% were female (*n* = 169). A significant proportion held a bachelor's degree (*n* = 204, 89.5%) with varying years of professional experience (24.5% more than 5 years’ experience). Nearly one-third (*n* = 85, 37.3%) were pharmacists or clinical pharmacists (Table 1).

**Table 1.** Sociodemographic characteristics of the study sample (*n* = 228).

Parameter	Frequency	(%)
**Age (years)**		
o 20–25	142	62.3
o 26–35	48	21.1
o 36–45	23	10.1
o 46–60	13	5.7
o More than 60	2	0.9
**Gender**		
o Male	59	25.9
o Female	169	74.1
**Profession**		
o Physician	19	8.3
o Pharmacist	85	37.3
o Nurse	51	22.4
o Other healthcare professionals	73	32.0
**Years of experience**		
o 1–2 years	147	84.5
o 3–5 years	25	11.0
o 6–10 years	27	11.8
o 11–20 years	18	7.9
o More than 20 years	11	4.8
**Educational level**		
o Bachelor degree	204	89.5
o Master degree	18	7.9
o PhD or equivalent	8	2.6

The study showed that most healthcare professionals in the sample exhibit low awareness of RSV or RSV vaccines. Specifically, only 43.0% (*n* = 98) heard about the RSV, and 28.1% (*n* = 64) were knowledgeable about the FDA-approved RSV vaccines. Participants’ perceptions regarding RSV and its vaccines were investigated, revealing significant insights (Table 2). More than half of respondents (*n* = 134, 58.8%) were unsure about the efficacy of RSV vaccines in preventing severe infections and didn't recognise that RSV is a common cause of hospitalisation or death in certain adult populations (*n* = 93, 40.8.0%). Nonetheless, most participants thought that they needed awareness regarding RSV vaccines. Although, most of the respondents will advise their patient to get the vaccine if it is safe and freely available (*n* = 208, 91.2), one third of them (*n* = 79, 34.7) were confident or somewhat confident in the vaccine's safety and effectiveness. Alarmingly, 96.9% of participants (*n* = 221) acknowledged the need for greater awareness and more information concerning the safety of RSV vaccines.

**Table 2.** Participants’ awareness, perceptions, and respondents practice regarding RSV and RSV vaccines (*n* = 228).

Questions/statement	YES Frequency (%)
Have you heard of the RSV?	98 (43.0)
I am aware about the FAD approved RSV vaccines	64 (28.1)
Are you able to distinguish RSV infection based on patients’ symptoms?	33 (14.3)
Are you very confident or somewhat confident in the safety and efficacy of RSV vaccines?	79 (34.7)
I believe my patients need RSV vaccines.	194 (85.1)
I am not sure about the efficacy of RSV vaccines in preventing severe infections	134 (58.8)
I am aware that RSV infection can cause hospitalisation or death in some patients	93 (40.8)
Did you provide care for at least one RSV patient?	45 (19.6)
Do you check the presence of RSV infections in patients with respiratory tract infections?	49 (21.3)
I will advise the patient to get the RSV vaccine	208 (91.2)
Awareness regarding RSV vaccines is needed	221 (96.9)
Information regarding the safety of RSV vaccines are needed	221 (96.9)
Test of RSV is rarely done because of the lack of treatment	56 (24.6)

In terms of clinical practices, the majority of respondents (*n* = 183, 79.8%) did not provide care to any RSV case. Furthermore, just 14.5% of respondents expressed confidence in their ability to distinguish RSV from other respiratory tract infections based on patient symptoms, and only 21.5% (*n* = 49) checked for the presence of RSV infections in patients who have respiratory tract infections. Additionally, 24.6% of respondents (*n* = 56) indicated that testing for RSV is infrequently performed due to the absence of effective treatments. Table 2.

Participants perceived several barriers to RSV vaccination if the vaccines were available, the most prominent being the patient's concerns about its safety (*n* = 194, 84.3%). A notable barrier was the potential out of pocket expenses (*n* = 185, 80.4%). The unwillingness of patients to have both RSV and influenza vaccines on a yearly basis (*n* = 81, 35.2%) presents a considerable barrier (Figure 1). Despite these barriers, a substantial proportion of respondents (194, 85.1%) affirmed that their patients require RSV vaccines.

Table 3 summarises the general knowledge responses of the study participants concerning RSV and its vaccines, with the mean score of knowledge being 3.5 (SD = 3.9). Less than half of respondents know that this virus usually causes mild, cold-like symptoms (*n* = 98, 42.6%), can cause serious illness in infants, some young children, and other adults (*n* = 106, 46.1%), or can spread through coughing, sneezing and contaminated surfaces (*n* = 104, 45.2%).

**Table 3.** Participants’ knowledge about RSV and RSV vaccines (*n* = 228).

Questions	Correct answer	Percent answered correctly *n* (%)
RSV usually causes mild, cold-like symptoms	True	98 (42.6)
RSV can cause serious illness in infants, some young children, and other adults	True	106 (46.1)
RSV can spread through coughing sneezing and contaminated surfaces	True	104 (45.2)
Transmission of the disease starts in the fall semester and peaks in winter	True	88 (38.3)
RSV is not a bacterial infection	True	206 (89.6)
Adults aged 75 years and older should receive a single dose of the RSV vaccine	True	54 (23.7)
Adults aged 60–74 years at increased risk of severe RSV disease should receive a single dose	True	71 (30.9)
RSV and influenza had similar hospitalisation rates in patients older than 50 years	True	38 (16.5)
RSV infection may result in hospitalisation or death	True	93 (40.4)

Only 38.3% of respondents (*n* = 88) answered correctly that the transmission of the disease starts in the fall semester and peaks in winter. Interestingly a significant proportion know that RSV is not a bacterial infection (*n* = 206, 89.6%). Additionally, 23.7% of participants identified that adults aged 75 years and older should receive a single dose of the RSV vaccine, while 31.1% indicated that adults aged 60–74 years at increased risk of severe RSV disease should also receive a single dose.

Linear regression analysis for factors influencing associated with participants’ knowledge (Table 4) showed that two of the studied demographic factors had a significant association with healthcare professionals’ knowledge regarding RSV and RSV vaccines (*p* > 0.05), namely educational level (having Master or PhD), and being physician or pharmacist.

**Table 4.** Assessment of factors associated with participants’ knowledge about RSV and RSV vaccines (*n* = 228).

Parameter	Knowledge score			
	Beta^a^	*p*-value^a^	Beta^b^	*p*-value^b^
Gender (female)	0.037	0.576	–	–
Age (more than 35 years)	0.368	0.089	0.027	0.628
Educational level (Master, PhD)	0.318	0.064	0.177	0.004*
Profession (physician or pharmacist)	−0.499	0.000	−0.417	0.000*
Experience (more than 5 years)	0.331	0.005	0.104	0.181

^a^ Using simple linear regression, ^b^Using multiple linear regression. *Significant at 0.05 significance level.

## 4. Discussion

This study represents the first comprehensive assessment of knowledge and practices related to the forthcoming RSV vaccine among healthcare professionals in Jordan. A notable majority of participants displayed an accurate understanding of the vaccine's availability and the transmission dynamics of RSV. In contrast, findings from a similar study conducted among general practitioners in Italy revealed a concerning lack of satisfactory understanding of RSV and inconsistent perceptions of risk, particularly regarding infections in the elderly population (Congedo et al., 2024).

The findings of this study reveal critical gaps in healthcare professionals’ knowledge, awareness, and clinical practices regarding respiratory syncytial virus (RSV) and its vaccines. Overall, the study highlights the need for improved education and awareness campaigns targeting healthcare professionals to enhance their understanding of RSV and its associated vaccination strategies (Papagiannis et al., 2024).

One of the most striking findings is the low level of awareness and knowledge about RSV among the study participants. Only 43.0% of healthcare professionals had heard of RSV, and a significantly lower proportion (28.1%) were knowledgeable about the FDA-approved RSV vaccines. This suggests a critical gap in both basic understanding of RSV and awareness of its preventive measures among healthcare professionals. These findings are concerning given that healthcare professionals play a crucial role in diagnosing, treating, and advising patients on vaccination options (Holford et al., 2024).

A substantial proportion of healthcare professionals (58.8%) were unsure about the efficacy of the RSV vaccine in preventing severe infections, and 61.0% did not recognise RSV as a common cause of hospitalisation in certain adult populations. This lack of awareness could potentially hinder the proper identification of at-risk individuals and delay appropriate interventions (Alfano et al., 2024). Furthermore, while many participants expressed the need for greater awareness about the safety and efficacy of RSV vaccines, this sentiment suggests a gap in current education efforts, despite the recognised need for vaccination in at-risk populations (Sinuraya et al., 2024).

Despite acknowledging the importance of RSV vaccination, healthcare professionals identified several barriers that may hinder the widespread adoption of the vaccine, should it become available. The most prominent barriers included patient concerns about vaccine safety (84.3%) and out-of-pocket expenses (80.4%). These concerns reflect broader challenges in healthcare delivery, such as vaccine hesitancy and the financial burden of vaccination, particularly in resource-limited settings (Nandi & Shet, 2020). Interestingly, the reluctance of patients to receive both the RSV and influenza vaccines on a yearly basis (35.2%) also emerged as a significant barrier, highlighting the challenge of achieving high vaccination coverage in a population already wary of frequent vaccinations (WHO, 2024). Medical practitioners may encounter patients who have misunderstandings or unfounded concerns about the safety of vaccines due to the rise in social media and disinformation. This can lead to skepticism even among medical professionals, particularly if they have been exposed to misleading information. Furthermore, medical professionals may be concerned about vaccine safety due to the possibility of adverse events. Despite the fact that most vaccines have been proven to be safe, the frequency of rare side effects may make medical professionals more cautious when discussing immunizations with patients.

Some medical professionals may be concerned about the long-term effects of more recent vaccines, particularly if they have only recently been put on the market. The cautious approach is often founded on the need for more thorough long-term safety data to fully understand the potential concerns, even when short-term trials show efficacy and safety (Nandi & Shet, 2020). Particularly if they work in environments where vaccine skepticism or reluctance is prevalent, healthcare workers are regularly influenced by the opinions and concerns of their peers. Their peers may have an impact on their personal beliefs, or they may grow reluctant to recommend vaccinations to patients.

The identification of these barriers underscores the importance of developing targeted strategies to address patient concerns and improve vaccine accessibility, including financial support and public education campaigns to increase confidence in vaccine safety (Tuckerman et al., 2022).

The study also revealed that healthcare professionals’ clinical practices in diagnosing and managing RSV infections are suboptimal. The majority of respondents (79.8%) reported not providing care to any patients with RSV, which may be attributed to the relatively low awareness of RSV as a significant cause of respiratory infections in both children and adults (Michelin et al., 2024). Additionally, 84.9% of respondents were unable to distinguish RSV from other respiratory tract infections, and only 21.3% regularly checked for RSV in patients with respiratory symptoms. These findings suggest that RSV may often be underdiagnosed, leading to missed opportunities for early intervention, particularly in vulnerable populations such as the elderly and infants (CDC, 2024).

Interestingly, nearly one-quarter of respondents (24.6%) indicated that testing for RSV was infrequently performed due to the lack of effective treatments, which could contribute to diagnostic delays. This highlights a need for better integration of RSV testing in clinical protocols, particularly given the emerging availability of RSV vaccines (Topalidou et al., 2023).

The linear regression analysis indicated that healthcare professionals’ educational level and professional background (e.g. physicians and pharmacists) were associated with better knowledge about RSV and its vaccines. This finding suggests that certain demographic characteristics, such as formal education and professional training, may influence the level of awareness and knowledge about RSV. This underscores the need for tailored educational interventions that target specific groups within the healthcare workforce, ensuring that all healthcare professionals, regardless of their professional role, are adequately informed about RSV and the benefits of vaccination (Gobbo et al., 2023).

The findings from this study have significant implications for public health initiatives aimed at increasing awareness and vaccination coverage for RSV. Given the low level of knowledge among healthcare professionals and the identified barriers to vaccination, it is critical to develop comprehensive training programmes that not only educate healthcare professionals about RSV and its vaccines but also equip them with the tools to effectively communicate these topics to patients. Addressing common misconceptions, providing evidence-based information on vaccine safety and efficacy, and offering strategies to manage patient concerns will be essential in improving the uptake of RSV vaccines (de Koning et al., 2024).

Furthermore, public health campaigns should focus on raising awareness about the importance of RSV vaccination, particularly among high-risk groups such as older adults and individuals with underlying health conditions. These efforts should also consider the financial and logistical challenges that may prevent widespread vaccine adoption, advocating for policy changes that support vaccine accessibility and affordability (Essien & Dusetzina, 2023).

In this study, while we acknowledge the limitations of using convenience sampling, we took steps to minimise selection bias by ensuring that participants were selected from a variety of healthcare settings (e.g. hospitals, clinics, private practices) to increase the diversity of the sample. Additionally, we tried to include a broad range of healthcare professionals from different specialties and regions, though we recognise that this approach may still not fully capture the diversity of the larger population., while, distributing the survey via social media platforms like Facebook and WhatsApp is practical and can quickly reach a wide audience, it may not ensure equal representation across all healthcare professionals. To address this limitation, we tried to use professional networks, specific healthcare forums, and collaboration with healthcare institutions to reach a broader and more diverse group of professionals. In Future studies could aim to use more rigorous sampling techniques, such as stratified random sampling, to reduce selection bias and a more structured approach to sampling would help enhance the generalizability of the findings and ensure that the data reflects the diversity of healthcare professionals.

Nonetheless, the findings of this study can still provide valuable insights into the specific population sampled.

## 5. Conclusion

In conclusion, this study highlights significant gaps in healthcare professionals’ knowledge, awareness, and clinical practices regarding RSV and its vaccines. Despite recognising the need for better education and greater awareness, there are substantial barriers to vaccine adoption and effective management of RSV infections. Addressing these issues through targeted educational interventions, improving diagnostic capabilities, and reducing barriers to vaccination will be critical steps in enhancing the healthcare response to RSV and preventing its serious health consequences.
